# Perioperative incidence of airway obstructive and hypoxemic events in patients with confirmed or suspected sleep apnea - a prospective, randomized pilot study comparing propofol/remifentanil and sevoflurane/remifentanil anesthesia

**DOI:** 10.1186/s12871-018-0477-9

**Published:** 2018-01-27

**Authors:** Philipp Fassbender, Silja Bürgener, Ali Haddad, Marie-Therese Silvanus, Jürgen Peters

**Affiliations:** Klinik für Anästhesiologie und Intensivmedizin, Universität Duisburg-Essen and Universitätsklinikum Essen, Hufelandstr 55, D-45147 Essen, Germany

**Keywords:** Anesthesia, Complications, Hypoxemia, Monitoring, Sleep apnea

## Abstract

**Background:**

Obstructive sleep apnea (OSA) is a risk factor for perioperative complications but data on anesthesia regimen are scarce.

**Methods:**

In patients with established or strongly suspected OSA, we assessed in a prospective, randomized design the effects on nocturnal apnea-hypopnea-index (AHI) and oxygen saturation (SpO_2_) of propofol/remifentanil or sevoflurane/remifentanil based anesthesia. Patients were selected by a history for OSA and / or a positive STOP – questionnaire and received general anesthesia using remifentanil (12 μg/kg/h) combined either with propofol (4-6 mg/kg/h, *n* = 27) or sevoflurane (approx. 2.2 vol% endtidal, n = 27). AHI and SpO_2_ were measured during the nights before and after anesthesia.

**Results:**

There were no differences in AHI between anesthetic regimens nor between the pre- and postoperative nights (propofol: 8.6 h^− 1^ (median, CI: 3.6–21.9) vs. 7.9 h^− 1^ (1.8–28.8); *p* = 0.97; sevoflurane: 3.8 h^− 1^ (1.8–7.3) vs. 2.9 h^− 1^ (1.2–9.5); *p* = 0.85). Postoperative minimum SpO_2_ (propofol: 80.7% ± 4.6, sevoflurane: 81.6 ± 4.6) did not differ from their respective preoperative baselines (propofol: 79.6% ± 6.5; *p* = 0.26, sevoflurane: 80.8% ± 5.2; *p* = 0.39). Even in patients with a preanesthetic AHI > 15, nocturnal AHI remained unchanged postoperatively.

**Conclusion:**

Thus, in a cohort of patients with suspected or confirmed OSA undergoing surgery of moderate duration and severity neither the volatile agent sevoflurane nor the intravenous anesthetic propofol altered nocturnal AHI or oxygen saturation, when combined with the short acting opioid remifentanil.

**Trial registration:**

German Clinical Trials Register, DRKS00005824 retrospectively registered on 03/12/2014.

## Background

Obstructive sleep apnea (OSA) is a common sleep related breathing disorder [1, 2] and it is characterized by recurrent nocturnal episodes of hypopnea and apnea evoking decreases in peripheral arterial oxygen saturation (SpO_2_) and other sequelae [3, 4]. The prevalence of OSA among the general population is high [5] and it is estimated that 11.4% of men and 4.7% of women suffer from moderate to severe OSA [6–8]. In surgical patients, its prevalence is even greater with up to 22% [9].

Several studies and meta-analyses have shown that OSA is an independent risk factor for a variety of perioperative pulmonary complications such as aspiration pneumonia, acute respiratory distress syndrome, pulmonary emboli, and reintubation / mechanical ventilation [10, 11]. Unfortunately, the vast majority (i.e., 70–90%) of OSA patients presents undiagnosed before surgery [2, 12]. While the American Society of Anesthesiologists has published guidelines for the perioperative management of patients with OSA, recommendations for specific anesthetic regimen do not exist [13]. In fact, randomized studies addressing specific anesthetic regimen for the management of OSA patients are missing. Furthermore, it is unclear whether OSA patients need special monitoring or surveillance following anesthesia/surgery, which is particular relevant in an ambulatory setting [14].

Accordingly, in a cohort of patients with established or strongly suspected OSA, we prospectively compared in a randomized fashion the effects on nocturnal apnea-hypopnea-index (AHI) and SpO_2_ of the volatile anesthetic sevoflurane and the intravenous anesthetic propofol when combined with the short acting opioid remifentanil for surgery of moderate duration and severity. To determine whether anesthetic regimens altered the incidence of obstructive and hypoxemic events variables were assessed both during the pre- and postoperative nights. Specifically, we tested the null hypotheses that nocturnal AHI and minimum SpO_2_ did not differ between 1) anesthetic regimens, and 2) preoperative vs. postoperative nights.

## Methods

After IRB approval and study registration (German Clinical Trials Register number DRKS00005824) we screened and enrolled after informed consent adult elective surgery patients with an established or suspected diagnosis of OSA, as defined by a positive prior overnight polysomnography and/or ≥2 positive STOP-criteria [15]. We enrolled patients undergoing surgery of moderate duration and severity which might be performed also in an ambulatory setting. Briefly, the STOP-questionnaire contains four questions (Do you *S*nore loudly? Do you often feel *T*ired, fatigued, or sleepy during daytime? Has anyone *O*bserved you stop breathing during your sleep? Do you have or are you being treated for high blood *P*ressure?), and positive answers to two or more questions classify patients at high risk for OSA.

OSA patients already on nocturnal continuous positive airway pressure (CPAP) therapy were excluded to avoid withdrawal of an established therapy solely for the purpose of this study. Further exclusion criteria were planned airway surgery, any procedure likely requiring postoperative mechanical ventilation, patients undergoing regional anesthesia, and a patient’s refusal to study participation.

The main types of surgery in our patients were eye surgery (including eye enucleation) (16 in the propofol group, 12 in the sevoflurane group), musculoskeletal surgery (7 and 6, respectively), and gynecology/urology surgery (3 and 6, respectively). The types of surgery, including the positioning of the patients, were not different between the groups.

### Measurements

In the night preceding surgery/anesthesia patients were instrumented with a portable polygraphy device (SomnoLab2, Weinmann, Hamburg, Germany) recording airflow (nasal pressure sensor), thoracic and abdominal respiratory excursions (piezoelectric measurement), SpO_2_ (pulse oxymetry), and body position (3-dimensional accelerometer). The device was programmed to start the recordings at 10 p.m. and to terminate recordings at 6 a.m. the next morning. No sedatives were given during the preoperative and postoperative nights, nor for pharmacological premedication. All recordings were performed in the respective patients´ surgical ward room.

For anesthesia, standard monitors (multi-lead electrocardiogram, non-invasive blood pressure (oscillometry), pulse oxymetry, and neuromuscular transmission monitoring by ulnar nerve stimulation and accelerometry (TOF-Watch, Essex Pharma GmbH, Munich, Germany) were applied.

Patients were then randomly (computer generated list with envelope drawing) assigned to receive either propofol/remifentanil or sevoflurane/remifentanil anesthesia. Anesthesia was induced in a standardized fashion using etomidate (0.3 mg kg^− 1^), remifentanil infusion (60 μg kg^− 1^ h^− 1^), and rocuronium (0.6 mg kg^− 1^). Following endotracheal intubation, anesthesia was maintained with either propofol (4–6 mg kg^− 1^ h^− 1^) and remifentanil (12 μg kg^− 1^ h^− 1^) or with sevoflurane (approx. 2.2 vol% endtidal, i.e., 1 MAC age adjusted) in air and remifentanil (12 μg kg^− 1^ h^− 1^). At the end of surgery patients were extubated when they were able to follow simple commands and the train-of-four-ratio had reached at least 0.9. Following extubation, patients were transferred to a post anesthesia care unit (PACU) and oxygen and intravenous morphine were administered at the discretion of the postoperative care team. Patients were discharged from the PACU when meeting the ASA’s PACU discharge criteria [16]. No patient required oxygen by mask or cannula upon discharge. On the first evening of anesthesia, the polygraphy device was applied again to assess any nocturnal postoperative obstructive or hyoxemic events.

All study personal was blind as to the results of the preoperative and postoperative measurements and anesthetic regimens used.

### Data analysis

Recorded data derived from the preoperative and postoperative nights were transferred to a computer and analyzed off-line by one of the investigators blind as to the anesthetic regimen using a dedicated software (SomnoLab®, Weinmann, Hamburg, Germany) and results were checked for artifacts and plausibility. An obstructive apneic event was defined as a ≥ 90% decrease in the flow amplitude compared to baseline with a duration ≥10 s in the presence of inspiratory attempts as seen by prevailing respiratory excursions. Hypopnea was defined as a ≥ 50% decrease in the flow amplitude compared to baseline concomitant with a ≥ 3% decrease in SpO_2_ and a duration ≥10 s.

Nocturnal apnea-hypopnea-index (AHI) was then calculated as the number of apnea/hypopnea events per hour. Any detection of an increased AHI in patients without a pre-existing diagnosis of OSA was reported to the respective patients before their hospital discharge and they were recommended to undergo further sleep studies.

### Statistical analyses

Given the absence of any prospective studies randomly comparing the effects of different general anesthesia regimens on postoperative AHI, we performed a sample size estimation on a surrogate variable, i.e., the differential effects of propofol and the volatile anesthetic isoflurane on upper airway genioglossus muscle activity, the key upper airway dilator mechanism [17]. Here, activity under propofol had decreased by approx. 70% compared to isoflurane. Since isoflurane increases airway critical closing pressure and evokes transient airway obstruction [18] we speculated that a total sample size of 54 patients would provide with an 80% power the ability to detect a substantial difference in AHI between anesthetics with an α error p of 5%.

Analyses were performed using Graphpad Prism® (GraphPad Software, La Jolla, CA, USA). We tested for normal distribution using the D’Agostino-Pearson normality test. Normally distributed data are presented as means ± standard deviation (SD) and non-normally distributed data are presented as median ((95% confidence interval of the median (CI)). The Mann-Whitney U and Chi^2^-tests were used for calculating differences in variables between the two anesthetic regimens. Differences in values of variables between the respective pre- and postoperative nights were calculated using the Student’s t-test for paired samples or the Wilcoxon matched pairs test, as appropriate. An a priori alpha error p of less than 0.05 was assumed as statistically significant.

## Results

**Fig. 1:**
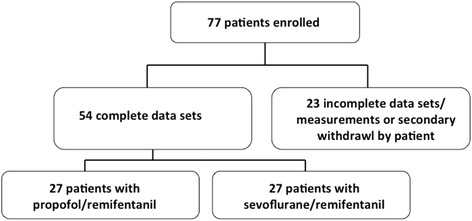
Flow chart of patient enrollment and data analysis

The study flow chart is depicted in Fig. 1. Seventy-seven patients gave consent to participate and were instrumented for the preoperative nocturnal measurements. Of those patients, 23 had to be excluded after study enrolment because of loss of data acquisition (involuntary or voluntary removal of sensors) or secondary withdrawal of consent, leaving 54 patients for analysis. Twenty-one (38%) patients reported a sleep-laboratory established diagnosis of OSA (but did not receive CPAP-therapy) and 33 (62%) patients fulfilled two or more positive STOP-criteria.

Twenty-seven patients received propofol/remifentanil anesthesia and another 27 patients sevoflurane/remifentanil anesthesia. Anesthesia duration averaged 97 min ± 43 (SD) and included breast cancer surgery, intervertebral disc surgery, eye/ENT surgery, and urologic surgery. The patients’ demographic data are shown in Table 1.

**Table 1:** Characteristics of patients receiving Propofol/remifentanil or Sevoflurane/remifentanil anesthesia

	Propofol/remifentanil anesthesia	Sevoflurane/remifentanil anesthesia	*p* - value
n	27	27	
Sex (male/female), n	18/9	16/11	
Age (years)	61.1 ± 13	59.5 ± 10	ns*
Body-Mass-Index (kg m^− 2^)	30.3 ± 7.2	33.1 ± 5.1	ns
Anesthesia duration (min)	85.3 ± 42	109.5 ± 42	ns
PACU§ opioid dose (morphine equivalent, mg)	9.5 ± 4.4	11.5 ± 6.4	ns

Data are presented as numbers or means ± SD

*ns not significant

§*PACU* post operative care unit

### Effects of the anesthetic regimens

**Fig. 2:**
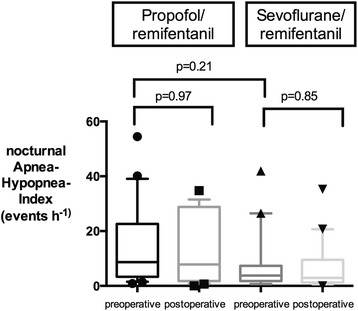
Nocturnal Apnea-Hypopnea-Index (AHI) in patients undergoing propofol/remifentanil (left panel) or sevoflurane/remifentanil (right panel) anesthesia, as assessed during the nights preceding and following surgery. Data are presented as box plots with median (center line), 25th and 75th percentile (box), and 10th and 90th percentile (whiskers), respectively. Outliers are presented as individual symbols. There was no significant change in the incidence of postoperative nocturnal obstructive events in either group compared to the respective preoperative baseline

Results are displayed in Fig. 2. We did not find a statistically significant effect of the anesthetic regimen on postoperative nocturnal AHI. Preoperative nocturnal AHI in the propofol group was 8.6 h^− 1^ (CI: 3.6–21.9) and remained unchanged during the first postoperative night (7.9 h^− 1^ (1.8–28.8); *p* = 0.97). Patients receiving sevoflurane had a slightly lower nocturnal AHI both pre- and postoperatively (3.8 h^− 1^ (1.8–7.3) vs. 2.9 h^− 1^ (1.2–9.5); *p* = 0.85). However, these differences were not statistically significant (*p* = 0.21, Fig. 2).

**Fig. 3:**
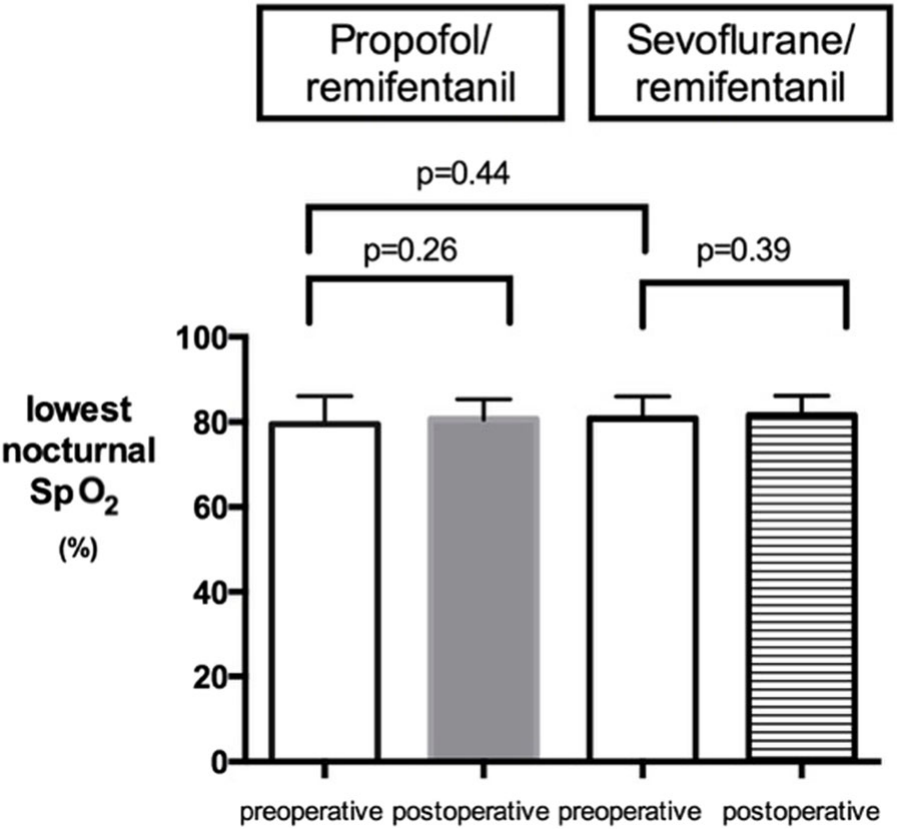
Minimum nocturnal arterial oxygen saturation (SpO_2_) in patients undergoing propofol/remifentanil (left panel) or sevoflurane/remifentanil (right panel) anesthesia, as assessed during the nights preceding and following surgery. Means±SD. The lowest postoperative nocturnal SpO_2_ did not differ from preoperative values in both groups nor between anesthesia regimens

Nocturnal oxygenation also did not change postoperatively compared to the preoperative night and we failed to find an effect of the anesthetic regimen (Fig.3). Preoperative minimum nocturnal SpO_2_ in the propofol group was 79.6% ± 6.5 and averaged 80.7% ± 4.6 postoperatively (*p* = 0.26). In the sevoflurane group, minimum nocturnal oxygen saturation during the preoperative night averaged 81.6% ± 4.6 and was 80.8% ± 5.2 postoperatively (*p* = 0.39).

### Effects irrespective of the anesthetic regimen

To investigate whether anesthesia per se, irrespective of its specific regimen, has an influence on postoperative nocturnal AHI or SpO_2_ we also pooled the data derived from all patients and could not find any difference in values of variables between the preoperative and postoperative nights. Specifically, nocturnal AHI was 5.9 h^− 1^ (3.8–9.6) preoperatively and 4.9 h^− 1^ (2.4–10.5) postoperatively (*p* = 0.54), and minimum SpO_2_ was 80.2% ± 5.7 and 81.2% ± 4.6 (*p* = 0.12), respectively.

Even patients with an AHI ≥ 15 did not show a postoperative nocturnal increase in AHI or SpO_2_ decrease with respect to the anesthetic regimen or their preoperative night.

All patients were followed up on the first postoperative day by the respective anesthesiologist in charge of clinical care. None of our patients had post-operative respiratory complications, requiring endotracheal intubation, non-invasive ventilation, or unplanned ICU admission.

## Discussion

This study, to our knowledge, is the first that prospectively and randomly compares the effects of two anesthetic regimens generally deemed suitable in OSA patients. Our data show, that 1) there was no difference in variables between propofol/remifentanil or sevoflurane/remifentanil anesthesia, and 2) postoperative nocturnal AHI and minimum SpO_2_ were unchanged when compared to the patients preoperative night. Thus, both anesthesia regimens did not worsen nocturnal airway obstruction or increase hypoxemic episodes, at least following surgeries of moderate duration and severity that could have been performed also in an ambulatory setting.

While OSA clearly is a risk factor for postoperative complications (for review see [14]) the contribution of specific anesthetic regimen is unknown and randomized studies are missing. Our finding, that neither anesthetic regimen increased postoperative nocturnal obstructive and hypoxemic episodes is consistent with other studies in OSA patients following general anesthesia [19, 20]. In fact, in a study of 31 obese OSA and non-OSA patients undergoing desflurane/remifentanil anesthesia no postoperative alterations of nocturnal postoperative oxygen saturation were found compared to their preoperative baseline [19]. Likewise, in 58 patients undergoing either regional or general anesthesia with various agents (sevoflurane, desflurane, fentanyl, morphine, and/or hydromorphone) and receiving polysomnography for up to 7 postoperative nights, AHI and minimum SpO_2_ also did not significantly change during the first postoperative night [20]. These findings are also consistent with a retrospective analysis of 234 ambulatory surgical patients with polysomnography confirmed OSA which revealed no greater incidence of unanticipated hospital admission or other adverse events than in non-OSA case controls [21]. In contrast, Chung et al. found a small increase in AHI in patients with mild and moderate OSA but surprisingly not in patients with severe OSA [22]. Accordingly, while OSA patients are at greater postoperative risk than non-OSA patients it is not evident whether specific anesthesia regimens alter nocturnal AHI and oxygenation. Thus, our study shows that the two anesthetic regimens based on the inhalational anesthetic sevoflurane and the intravenous anesthetic propofol appear safe, at least for the type of surgeries and patient cohorts studied, and do not increase nocturnal obstructive events.

In our study, anesthesia regimens were strictly standardized, rather short acting drugs were used, and substantial residual neuromuscular blockade was ruled out before extubation. Most prior observational studies in OSA patients do not have such a high degree of standardization. In particular, if longer acting neuromuscular drugs are used in the absence of neuromuscular monitoring greater interference with nocturnal upper airway patency and oxygenation irrespective of the specific anesthetic drugs may not be excluded [23].

While the literature and guidelines provide little help in defining an appropriate duration of postoperative respiratory monitoring in patients with OSA, the first postoperative night is generally believed to constitute a particular risk for OSA patients [24] and, therefore, monitoring is usually applied during the first postoperative night only in OSA patients. The question remains whether measurements of AHI and minimum oxygenation during the first postoperative night in OSA patients are representative for their entire postoperative course. In fact, in patients undergoing polysomnography for up to 7 postoperative nights [20, 22] the frequency of sleep disordered breathing appeared to increase on the third postoperative night, raising the hypothesis that sleep disordered breathing, if anything, may not deteriorate until the third postoperative night. While this question has to be addressed, the latter data are consistent with the provocative speculation that OSA patients undergoing surgery/anesthesia of moderate duration do not deteriorate the night following anesthesia, but may require special monitoring or surveillance later.

Several limitations of our pilot study should be mentioned. First, the majority of our patients did not suffer from the most severe degree of OSA, due to the exclusion of OSA patients already on nocturnal home CPAP therapy. Even some patients with a preexisting sleep laboratory derived OSA diagnosis had a low AHI in our study. Intra-individual variability in OSA severity is well recognized and may be partly explained by day-to-day changes in evening leg fluid volume and overnight rostral fluid shift [25]. However, even in our patients with severe OSA AHI and SpO_2_ were not altered by either anesthetic regimen. Second, not all of our patients had a polysomnography established OSA diagnosis and the STOP-Questionnaire has a lesser sensitivity and specificity in identifying such patients [15]. However, this applies to both of our subcohorts and even patients with a high preoperative nocturnal AHI revealed an unchanged AHI the night following anesthesia. Third, we did not assess the doses of analgesics that our patients received after discharge from the PACU, what might be a potential confounder if these doses were significantly different between the groups. However, as the doses of opioids received in the PACU and the types and length of surgery did not differ between the groups, we are confident that unmeasured opioid intake did not affect results. Fourth, another potential confounder would be chronic alcohol consumption, as past and/or current alcohol consumption influences the prevalence and severity of obstructive sleep apnea [26, 27]. However, none of our patients revealed such a history on preanesthetic evaluation. Finally, the number of patients was small and different results may be observed in patients with more extensive surgeries, in particular abdominal or intrathoracic surgery. These issues must be specifically addressed in different, also very standardized studies.

## Conclusion

Neither propofol/remifentanil nor sevoflurane/remifentanil anesthesia worsened the incidence of postoperative nocturnal obstructive or hypoxemic events following medium duration surgery in a cohort of patients with established or strongly suspected OSA when compared to the preoperative night. Furthermore, there was no difference between these anesthetic regimens. Thus, while our data suggest that both anesthetic regimen are safe in patients with confirmed or suspected OSA undergoing anesthesia of moderate duration, they also support the view that these patients do not deteriorate below their preoperative baseline the night following anesthesia.
